# DISSEMINATION STRATEGIES: ALIGNMENT WITH GERI-ED

**DOI:** 10.1093/geroni/igz038.292

**Published:** 2019-11-08

**Authors:** Tim Platts-Mills, Tony Rosen, Rebecca Jackson Stoeckle, Kim Dash, Kristin Lees-Haggerty

**Affiliations:** 1 University of North Carolina, Chapel Hill, North Carolina, United States; 2 Weill Cornell Medical College, New York, New York, United States; 3 Education development Center, Waltham, Massachusetts, United States

## Abstract

The National Collaboratory to Address Elder Mistreatment’s model provides hospital emergency departments with training and tools to help recognize and respond to elder mistreatment. The Geriatric Emergency Department (GERI-ED) initiative, supported by The John A. Hartford Foundation and West Health, encourages hospitals to integrate best practices for older adults into their emergency departments by providing guidelines and options for accreditation (GED-A). Recognizing the importance of coordinating efforts to develop tools that can be shared and implemented widely, the Collaboratory has intentionally and strategically aligned itself with the GERI-ED by inviting members of the GED-A team to join the Collaboratory. In this presentation we will describe how aligning these two initiatives has the potential to achieve greater impact than either initiative alone. We will provide a case example of how the elder mistreatment care model is implemented in GERI-EDs, highlighting factors that facilitate and hinder model implementation in such settings.

